# Clinical significance of serum omentin-1 levels in patients with pancreatic adenocarcinoma

**DOI:** 10.1016/j.bbacli.2016.10.002

**Published:** 2016-10-15

**Authors:** Senem Karabulut, Cigdem Usul Afsar, Mehmet Karabulut, Halil Alis, Mehmet Abdussamet Bozkurt, Fatma Aydogan, Murat Serilmez, Faruk Tas

**Affiliations:** a Department of Medical Oncology, Institute of Oncology, Istanbul University, Istanbul, Turkey; b Department of Medical Oncology, Istanbul Education and Research Hospital, Istanbul, Turkey; c Clinic of General Surgery, Istanbul Bakırköy Dr. Sadi Konuk Education and Research Hospital, Istanbul, Turkey; d Department of Basic Oncology, Institute of Oncology, Istanbul University, Istanbul, Turkey

**Keywords:** Omentin, Diagnostic, Pancreatic adenocancer, Serum

## Abstract

**Background:**

Omentin is related with metabolic syndrome and obesity. Pancreatic adenocarcinoma (PA) is a lethal and obesity-linked malignancy. This study was conducted to investigate the serum levels of omentin in patients with PA and the relationship with tumor progression and known prognostic parameters.

**Material and methods:**

Serum samples were obtained from thirty-three patients on first admission before any treatment. Age, sex and body mass index (BMI) matched 30 healthy controls were included in the analysis. Both serum omentin levels were measured using enzyme-linked immunosorbent assay (ELISA).

**Results:**

The median age at diagnosis was 59 years (32–84 years). Twenty (61%) patients were men and the remaining were women. The most common metastatic site was liver in 23 patients with metastasis (n = 19, 83%). Thirty-nine percent of 23 metastatic patients who received palliative chemotherapy (CTx) were CTx–responsive. Median overall survival of the whole group was 41.3 ± 8.3 weeks [95% confidence interval (CI) = 25–58 weeks]. The baseline serum omentin levels were significantly higher in patients with PA than in the control group (p < 0.001). Serum omentin levels were significantly higher in patients with larger pathologic tumor size compared with smaller size (p = 0.03). Conversely, serum omentin concentration was found to have no prognostic role on survival (p = 0.54).

**Conclusion:**

Serum levels of omentin may have a good diagnostic role in patients with PA.

## Highlights

• We want to emphasize the fact that our article is important because it is the only one about omentin levels and pancreatic cancer relationship.

## 1 Introduction

Pancreatic adenocarcinoma (PA) is the eighth leading cause of cancer deaths in men and the ninth in women worldwide. The majority of these tumors (85%) are adenocarcinomas arising from the ductal epithelium. Approximately 48.960 patients are diagnosed with cancer of the exocrine pancreas annually in the United States, and almost all are expected to die from the disease due to its aggressive nature [1].

The link between high body mass, lack of physical activity, and PC risk has been illustrated in several studies [2], [3], [4]. Several molecular factors may play an important role in the association between obesity and cancer, including insulin resistance, aberrant insulin like growth factor expression, sex hormones disorder and adipocytokines [5]. Currently adipose tissue is considered as an active endocrine organ with metabolic and immune regulatory roles [6]. Adipose tissue secrets a variety of proteins, including adipokines. Furthermore, adipocytokines might cause the proliferation and growth of tumor cells, induce inflammation and anti-apoptosis pathways, which subsequently can prompt cancer metastasis [7].

Omentin-1 is a 34-kDa adipocytokine that is primarily secreted from stromal vascular cells of visceral adipose tissue and enhance insulin sensitivity and glucose metabolism in normal weight individuals. Omentin-1 is considered to play a role in inflammatory responses and cell differentiation, and also promotes apoptosis of cancer cells [8], [9], [10]. In prostate and colorectal carcinoma, serum omentin levels were found to be high and in renal cell carcinoma it is found to be decreased [11], [12], [13], [14]. In acute and chronic pancreatitis, the elevation in omentin levels was due to the anti-inflammatory effects of omentin and elevated omentin levels improved insulin resistance, caused a significant reduction in glucose levels [15]. In literature, there is limited data about omentin and cancer relationship. To our knowledge; our study is the only one in pancreatic carcinoma.

## 2 Material and methods

### 2.1 Patients' characteristics

The data of 33 patients with histologically confirmed diagnosis of PA were recorded from their medical charts. The staging of metastatic patients was done by using computed tomography (CT), magnetic resonance imaging (MRI), and positron emission computed tomography (PET/CT) scan. Patients were staged according to the International Union Against Cancer TNM classification.

Chemotherapy (CTx) was given to the majority of the patients with metastatic disease (n = 20, 61%). Regimens of single or combination CTx were selected based on the performance status of patients and extension of the disease. CTx schemes were applied as follows: combination of gemcitabine with platinum or capecitabine (n = 6 and n = 3), or gemcitabine alone (n = 11). Response to treatment was determined by radiologically after 2–3 cycles of CTx according to revised RECIST criteria version 1.1. by the investigators and classified as follows: complete response (CR), partial response (PR), stable disease (SD), or progressive disease (PD). The tumor response after 2 months of CTx was used for statistical analysis. Follow-up programs of metastatic disease consisted of clinical, laboratory, and imaging by using a CT or MRI depending on which imaging methods were used at baseline and performed at 8-week intervals during CTx or every 12 weeks for no anticancer treatment. Patients with either PR or SD were classified as responders, and patients with PD were considered non-responders.

The possible prognostic variables were selected based on those identified in previous studies. Serum carcino-embryonic antigen (CEA) and carbohydrate antigen (CA) 19.9 levels were determined by microparticle enzyme immunoassay (Abbott Diagnostics, Chicago, IL). Serum erythrocyte sedimentation rate (ESR), lactate dehydrogenase (LDH) levels, albumin and, whole blood count assays were measured at presentation in our biochemical laboratory. Serum LDH activity was determined immediately after collection by the kinetic method on a Targa-3000 autoanalyzer (Pointe Scientific Inc., Lincoln Park, MI, U.S.A.) at 37 °C. The laboratory parameters were evaluated at diagnosis within the normal ranges of our institution.

For comparison of serum levels of omentin, age, sex and BMI matched 30 healthy controls were included in the analysis. Blood samples were obtained from patients with PA at first admission before any treatment. Institutional review board approval was obtained from each subject prior to the commencement of the study.

### 2.2 Measurement of serum omentin levels

This assay is a Sandwich ELISA based on: 1) capture of omentin-1 molecules in the sample by anti-omentin IgG and immobilization of the resulting complex to the wells of a microtiter plate coated by a pre-titered amount of anchor antibodies, 2) and the simultaneous binding of a second biotinylated antibody to omentin-1, 3) wash away of unbound materials, followed by conjugation of horseradish peroxidase to the immobilized biotinylated antibodies, 4) wash-away of free enzyme, and 5) quantification of immobilized antibody-enzyme conjugates by monitoring horseradish peroxidase activities in the presence of the substrate 3,3′,5,5′-tetra-methylbenzidine. The enzyme activity is measured spectrophotometrically by the increased absorbency at 450 nm, corrected from the absorbency at 590 nm, after acidification of formed products. Since the increase in absorbency is directly proportional to the amount of captured omentin-1 in the unknown sample, the concentration of omentin-1 can be derived by interpolation from a reference curve generated in the same assay with reference standards of known concentrations of omentin-1. The color development is stopped and the intensity of the color is measured using an automated ELISA reader (ChroMate, 4300 Microplate Reader, Palm City FL, USA). The results were expressed as ng/mL.

### 2.3 Statistical analysis

Continuous variables were categorized using median values as cut-off point. For group comparison of categorical variables, Chi-square tests or One-Way Anova tests were used and for comparison of continuous variables, Mann–Whitney *U* test or Kruskal-Wallis tests was accomplished. Overall survival (OS) was calculated from the date of first admission to the clinics to disease-related death or date of last contact with the patient or any family member. Kaplan-Meier method was used for the estimation of survival distribution and differences in OS was assessed by the log-rank statistics. All statistical tests were carried out two-sided and a p value ≤ 0.05 was considered statistically significant. Statistical analysis was carried out using SPPS 21.0 (SPSS Inc., Chicago, IL., USA) software.

## 3 Results

From February 2010 to July 2013, 33 patients with a pathologically confirmed diagnosis of PA were enrolled in this study. The baseline histopathological characteristics and the demographic characteristics of the patients are listed in Table 1. The median age at diagnosis was 59 years, range 32–84 years; majority of the patients in the group were men (n = 20, 61%). The tumor was located in the head of pancreas in 21 (64%) patients. Thirty-nine percent of 23 metastatic patients who received palliative CTx were CTx-responsive. The most common metastatic site was liver in 23 patients with metastasis (n = 19, 83%). Surgery was performed in 8 (24%) patients; 5 (15%) patients underwent pancreaticoduodenectomy and 3 (9%) patients had palliative surgery.

The levels of serum omentin assays in patients with PA and healthy controls are shown in Table 2. The baseline serum omentin levels were significantly higher in patients with PA than in the control group (median 9.57 v. 1.61 ng/mL, p < 0.001).

Table 3 shows the correlation between the serum levels omentin of and clinico-pathological factors. Serum omentin levels were significantly higher in large pathologic tumor size compared with small pathologic tumor size (p = 0.03).

**Fig. 1:**
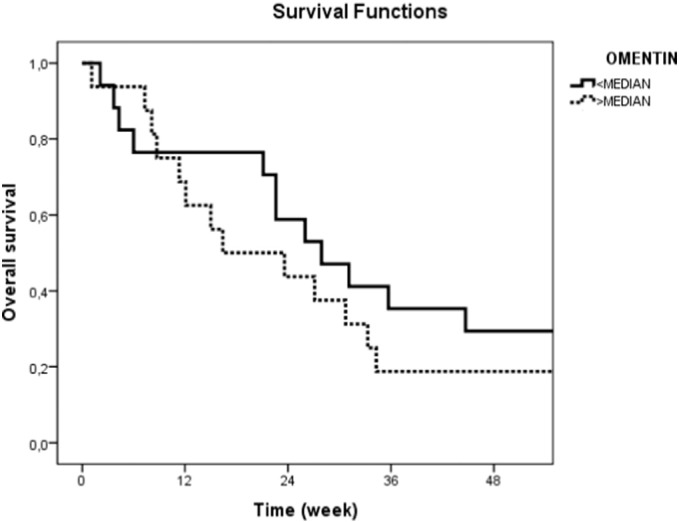
Overall survival curves in pancreatic cancer patients according to serum omentin levels (p = 0.54).

The median follow-up time was 26.0 weeks (range: 1.0–184.0 weeks). At the end of the observation period, thirty-two patients (97%) were dead. Median OS of the whole group were 41.3 ± 8.3 weeks [95% confidence interval (CI) = 25–58 weeks]. While 1-year OS rates were 24.2% (95% CI = 9.5–38.9). Older age, worse performance status, metastatic disease, lack of liver metastases and the CTx-unresponsiveness were found to be significant prognostic factors (p = 0.008, p = 0.002, p = 0.008, p = 0.02, and p = 0.03, respectively). However, serum omentin levels had no significantly effect on OS rates (p = 0.54) (Table 4 and Fig. 1).

## 4 Discussion

Although omentin-1 levels were found to be changed in some cancers, its possible clinical significance has remained unclear in patients with pancreatic cancer. Only a few studies have been previously performed. Both colorectal and pancreatic cancers are related with obesity, metabolic syndrome and BMI. Recently clinical studies show that cancers such as liver [10], prostate [12] and colorectal [11], [13], [16] are associated with increases in omentin serum levels independent of various factors such as BMI, glucose, lipid parameters, disease differentiation [16]. In a new study, higher omentin concentrations were associated with a higher colorectal cancer risk independent of obesity [16].

To the best of our knowledge, there are no additional studies directly associating the anti-inflammatory and tumor-suppressing effects of omentin on other cancers. There is also no data in literature about the relationship of serum omentin-1 levels and PA. There is only limited data about pancreatitis and omentin levels. The elevation in omentin levels in early stage of pancreatitis was found; it caused insulin resistance and reduction in glucose levels [15].

In our study, we showed that in patients with PA, serum omentin-1 levels were elevated. Serum omentin levels were significantly higher in large pathologic tumor (≥ 4 cm) size compared with small pathologic tumor size. This finding is really interesting.

In cancer studies, omentin was suggested to promote cancer cell growth by triggering genomic instability and PI3K/Akt (phosphatidylinositol-3 kinase downstream effector) signaling pathways and the cancer-promoting effects of omentin was independent of its abilities to regulate obesity-induced metabolic risk [11], [17], [18], [19]. Omentin may also show a number of effects reflecting cellular immune responses. In the area of oncologic treatments, immunooncology is a promising topic. Maybe, omentin shows its effects as an antiinflammatory marker.

In conclusion, the present study revealed that serum levels of omentin-1 were only a diagnostic marker in pancreatic cancer patients. However, its predictive and prognostic values were not determined. In addition, no correlation was observed in serum omentin level and response to chemotherapy. The small sample size of the present study may be considered as significant limitation and may have influenced these results. Further studies in a larger patient population are needed.

## Transparency document

Transparency document Image 1

## Figures and Tables

**Table 1.** Characteristics of the patients and disease.

Variables	n
No. of patients	33
Age (years)	
Median (range)	59 (32–84)
Gender	
Male/female	20/13
Performance status (PS)^a^	
0/1/2/3	4/19/5/4
Weight loss^a^	
Yes/no	26/4
Jaundice^a^	
Yes/no	9/22
Surgery type^b^	
Whipple surgery/palliative surgery	5/3
Pathologic tumor (pT) size^a^	
< Small (< 40 mm)/≥ large (≥ 40 mm)	14/14
Site of lesion	
Head/corpus-tail	21/10
Response to chemotherapy (CTx)	
Yes (PR + SD)/no (PD)	9/11
Metastasis	
Yes/no	23/10
Erythrocyte sedimentation rate (ESH)	
Normal (< 40/h)/high (> 40/h)	11/12
White blood cell count (WBC)^a^	
Normal (< 10.000/mm^3^)/high (> 10.000/mm^3^)	22/9
Hemoglobin (Hb)^a^	
Low (< 12 g/dL)/normal (> 12 g/dL)	12/19
Platelet count (PLT)^a^	
Low (< 150.000/mm^3^)/normal (> 150.000/mm^3^)	5/26
Lactate dehydrogenase (LDH)^a^	
Normal (< 450 IU/L)/high (> 450 IU/L)	21/8
Albumin^a^	
Low (< 4 gr/dL)/normal (> 4 gr/dL)	10/17
Carcinoembryonic antigen (CEA)^a^	
Normal (< 5 ng/mL)/high (> 5 ng/mL)	19/10
Carbohydrate antigen (CA 19.9)^a^	
Normal (< 38 U/mL)/high (> 38 U/mL)	7/22

a Patients with unknown data concerning the variables are not included in the analysis.

b In 10 patients with non-metastatic disease.

**Table 2:** The values of serum marker levels in pancreatic cancer patients and healthy controls.

	Patients (n = 33)		Controls (n = 30)		
Marker	Median	Range	Median	Range	p
Omentin (ng/mL)	9.57	3.62–219.48	1.61	0.80–4.98	< 0.001⁎

⁎ p ≤ 0.05.

**Table 3:** Results (median and range) of comparisons between the omentin marker assays and various clinical parameters.

Parameters	Marker assays		
	n	Omentin (ng/mL) Median (range)	p
*Age patients*			
Young (< 60)	18	8.19 (3.62–219.48)	0.40
Older (> 60)	15	10.48 (5.15–204.24)	

*Gender*			
Male	20	10.30 (3.62–219.48)	0.92
Female	13	9.27 (5.85–194.50)	

*PS*			
Good (0–1)	23	10.28 (3.62–219.48)	0.84
Worse (2–4)	9	9.57 (3.62–204.24)	

*Weight loss*			
Yes	26	9.93 (3.62–205.14)	0.79
No	4	74.69 (5.15–219.48)	

*Jaundice*			
Yes	9	7.49 (3.62–193.88)	0.45
No	22	10.30 (3.62–219.48)	

*Surgery*			
Yes	8	7.78 (5.26–205.14)	0.75
No	25	9.42 (3.62–219.48)	

*Localization*			
Head	21	8.59 (3.62–205.14)	0.44
Corpus-tail	10	10.60 (3.62–204.24)	

*pT size*			
Small (< 40 mm)	14	7.65 (3.62–193.88)	0.03⁎
Large (≥ 40 mm)	14	74.29 (3.62–205.14)	

*Metastasis*			
Yes	23	10.34 (3.62–219.48)	0.43
No	10	8.05 (3.62–193.88)	

*Liver metastasis*			
Yes	19	10.34 (5.26–219.48)	0.41
No	4	71.25 (3.62–188.14)	

*ESH*			
Normal	11	9.42 (3.62–219.48)	0.70
High	12	7.78 (5.37–205.14)	

*Hb*			
Low	12	10.48 (5.15–219.48)	0.08
Normal	19	7.13 (3.62–204.24)	

*WBC*			
High	9	7.78 (5.26–204.24)	0.88
Normal	22	10.30 (3.62–219.48)	

*PLT*			
Low	5	10.86 (6.63–219.48)	0.39
Normal	26	8.93 (3.62–205.14)	

*Albumin*			
Low	17	7.50 (3.62–194.50)	0.68
Normal	10	10.28 (5.15–219.48)	

*LDH*			
High	8	10.03 (5.42–204.24)	0.58
Normal	21	8.59 (3.62–219.48)	

*CEA*			
High	19	74.11 (5.42–219.48)	0.09
Normal	10	7.78 (3.62–205.14)	

*CA 19.9*			
High	22	9.95 (3.62–205.14)	0.50
Normal	7	8.59 (5.15–219.48)	

*Response to CTx*			
Yes (PR + SD)	8	7.78 (3.62–205.14)	0.71
No (PD)	7	7.41 (5.15–249.48)	

⁎ p ≤ 0.05.

**Table 4:** Univariate analyses of overall survival.

Parameters	Overall survival Median (± SD) (weeks)	p
*Age patients*		
Young	58.3 (13.1)	0.008⁎
Older	21.8 (6.6)	

*Gender*		
Male	49.9 (12.6)	0.21
Female	29.0 (7.5)	

*PS*		
Good	53.6 (10.9)	0.002⁎
Worse	15.6 (3.6)	

*Weight loss*		
Yes	36.7 (6.6)	0.34
No	74.5 (41.5)	

*Jaundice*		
Yes	41.6 (18.8)	0.46
No	41.9 (7.8)	

*Localization*		
Head	48.3 (11.8)	0.54
Corpus-tail	34.4 (10.4)	

*pT size*		
Small	42.1 (9.4)	0.37
Large	36.4 (8.9)	

*Metastasis*		
Yes	26.5 (5.9)	0.008⁎
No	76.7 (20.3)	

*Liver metastasis*		
Yes	30.0 (6.8)	0.02⁎
No	9.5 (4.6)	

*ESR*		
High	34.3 (7.7)	0.24
Normal	43.9 (12.0)	

*Hb*		
Low	41.1 (11.5)	0.66
Normal	32.1 (7.0)	

*WBC*		
High	38.2 (12.2)	0.67
Normal	34.5 (7.2)	

*PLT*		
Low	27.5 (9.0)	0.59
Normal	37.2 (7.1)	

*Albumin*		
Low	30.9 (8.8)	0.79
Normal	32.8 (8.7)	

*LDH*		
High	24.5 (12.2)	0.06
Normal	38.3 (6.8)	

*CEA*		
High	30.1 (9.4)	0.66
Normal	36.8 (7.7)	

*CA 19.9*		
High	32.5 (6.0)	0.63
Normal	40.8 (16.0)	

*Response to CTx*		
Yes	48.1 (11.4)	0.03⁎
No	23.1 (8.9)	

*Omentin*		
< Median	41.3 (8.8)	0.54
> Median	42.1 (14.6)	

⁎ p ≤ 0.05.
